# Histiocytic sarcoma combined with acute monocytic leukemia: a case report

**DOI:** 10.1186/s13000-015-0350-9

**Published:** 2015-07-19

**Authors:** Jiangning Zhao, Xiaoqing Niu, Zhao Wang, Huadong Lu, Xiaoyan Lin, Quanyi Lu

**Affiliations:** Department of Hematology, Zhongshan Hospital of Xiamen University, Xiamen, 361004 Fujian China; Department of Pathology, Zhongshan Hospital of Xiamen University, Xiamen, 361004 Fujian China; Clinical Laboratories, Zhongshan Hospital of Xiamen University, Xiamen, 361004 Fujian China

**Keywords:** Histiocytic sarcoma, Acute monocytic leukemia, Relationship

## Abstract

**Background:**

Histiocytic sarcoma (HS) is a rare malignant tumor. Underlying or associated disorders have been reported in some patients with HS. We herein report a very rare case of HS combined with acute monocytic leukemia (AMoL).

**Case presentation:**

A 62-year-old man presented with systemic lymph node enlargement and pancytopenia in August 2012. Bone marrow (BM) aspirate showed abnormal hematopoiesis with 3 % blast, but no obvious abnormalities on flow cytometric immunophenotyping. A BM cytogenetic study and fluorescence in situ hybridization revealed a 46, XY karyotype and no myelodysplastic syndrome-associated features, respectively. A right cervical node biopsy showed disrupted node structure with diffuse pleomorphic neoplastic cells that were positive for cluster of differentiation (CD) 68, MAC387 and lysozyme, but negative for CD1a, CD21, CD30, S100, and T-cell, B-cell, and myeloid lineage markers. The patient was diagnosed with HS and treated with 8 courses of CHOP chemotherapy. After 4 courses, total-body FDG-PET imaging showed partial remission and disappearance of abnormal hematopoiesis in the BM, but 2 % blasts remained. Lymphadenopathy and pancytopenia recurred 1 month after the his last chemotherapy dose. He became resistant to second-line chemotherapy, with gradually increasing leukocytes, up to 50 % blasts in BM in December 2013, and abnormal cells positive for CD117, CD13, CD33, HLA-DR, CD34, CD11c, CD38, and myeloperoxidase. He was diagnosed with acute monocytic leukemia (AMoL-M5), and treated by CAG regimen + decitabine, but died of severe pneumonia and hepatic failure.

**Conclusion:**

To our knowledge, this is the first case of HS combined with AMoL. The coexistence of these two neoplasms was shown by the lymph node biopsy findings and BM myeloid markers. The patient had a transient response to chemotherapy and a poor prognosis. Whether these two neoplasms were related is unclear; however, if so, we suspect the combination might be caused by a malignant transformation of a promonocyte or stem cell, upstream of histiocytes and monocytes.

## Background

Histiocytic sarcoma (HS) is an extremely rare malignant neoplasm, accounting for less than 1 % of all hematolymphoid neoplasms [1]. It is believed to be derived from monocyte/macrophage lineage and shows morphologic and immunophenotypic features of mature tissue histiocytes. According to the 2001 and 2008 World Health Organization (WHO) classifications [1, 2], HS cells are immunohistochemically positive for one or more histiocytic markers such as cluster of differentiation (CD) 68 (KP1, PGM1), CD163, and lysozyme, but negative for CD1a, CD21, CD35, CD30, and T-cell, B-cell, and myeloid lineage markers. S100 can be positive but usually weak or focal. Ki67 is variable. With the development of immunohistochemical (IHC) techniques, most previous reported cases of HS are now generally recognized to be misdiagnosed examples of non-Hodgkin lymphomas, predominantly diffuse large B-cell lymphoma, or anaplastic cell lymphoma.

Interestingly, many reports have described the association of HS with other neoplasms, especially hematologic malignancies, the most common of which are lymphocytic leukemia/lymphomas, such as follicular lymphoma [3, 4], mantle cell lymphoma [5], mucosa-associated lymphoid tissue lymphoma [6], diffuse large B-cell lymphoma [7], acute lymphoblastic leukemia [8–11] and chronic lymphocytic leukemia [12], chronic myelomonocytic leukemia (CMML) [13], mediastinal germ cell tumors [14, 15], and idiopathic myelofibrosis [16]. Moreover, HS has been shown to share molecular or cytogenetic features with the associated neoplasms in most of these cases.

We herein report a case involving a 62-year-old man who was initially diagnosed with HS and later found to have acute monocytic leukemia (AMoL). To our knowledge, this is the first reported case of HS combined with AMoL.

## Case presentation

**Fig. 1:**
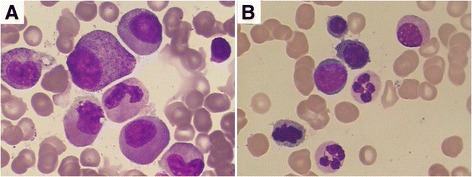
Comparison of cytology of bone marrow cells before and after treatment of HS (×1,000, Wright–Giemsa stain). **a** A bone marrow smear on first admission showed there were granulocytic predominance and no significant increase in monocytes. There was abnormal hematopoiesis in granulocyte, erythroid and megakaryocytic series in different extent with 3 % blasts. **b** A bone marrow smear after 4 courses of CHOP chemotherapy showed abnormal hematopoiesis was disappeared, although 2 % blasts remained

**Fig. 2:**
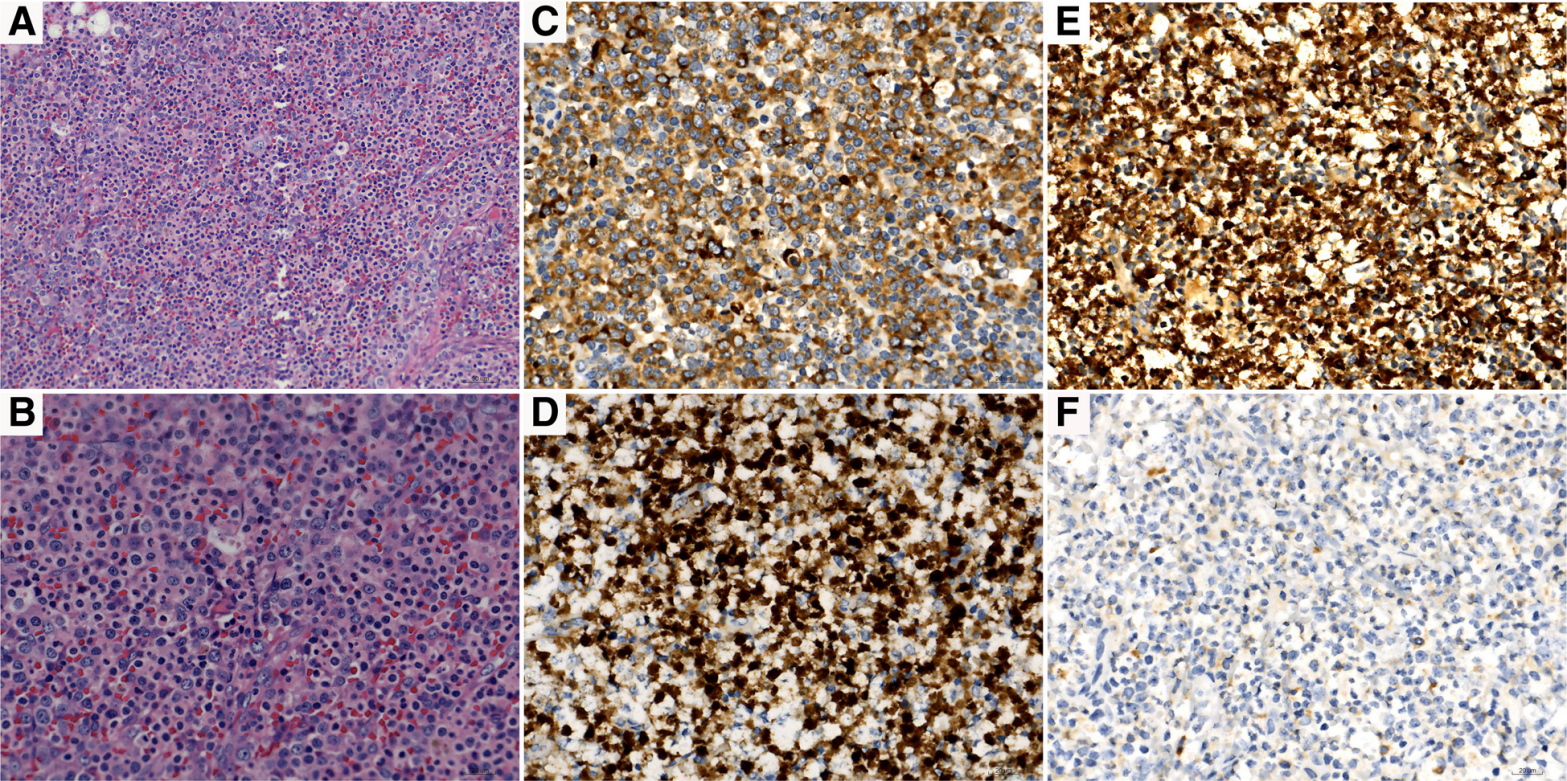
Histologic picture and immunohistochemistry panel of a right cervical lymph node. The node structure was disrupted with effacement by diffuse distribution of pleomorphic neoplastic cells (×200, H&E) (**a**), most of which were large, and round to oval in shape. Some neoplastic giant cells were observed. The cytoplasm was usually abundant, and the nuclei were generally large and round with a prominent nucleolus. The chromatin was hyperchromatic, coarse, and granular. Mitotic figures were easily observed. Remaining lymphoid follicles were found (×400, H&E) (**b**). IHC phenotype of neoplastic cells show positive for CD68 (**c**), MAC387 (**d**), lysozyme (**e**), but negative for myeloperoxidase (**f**)

**Fig. 3:**
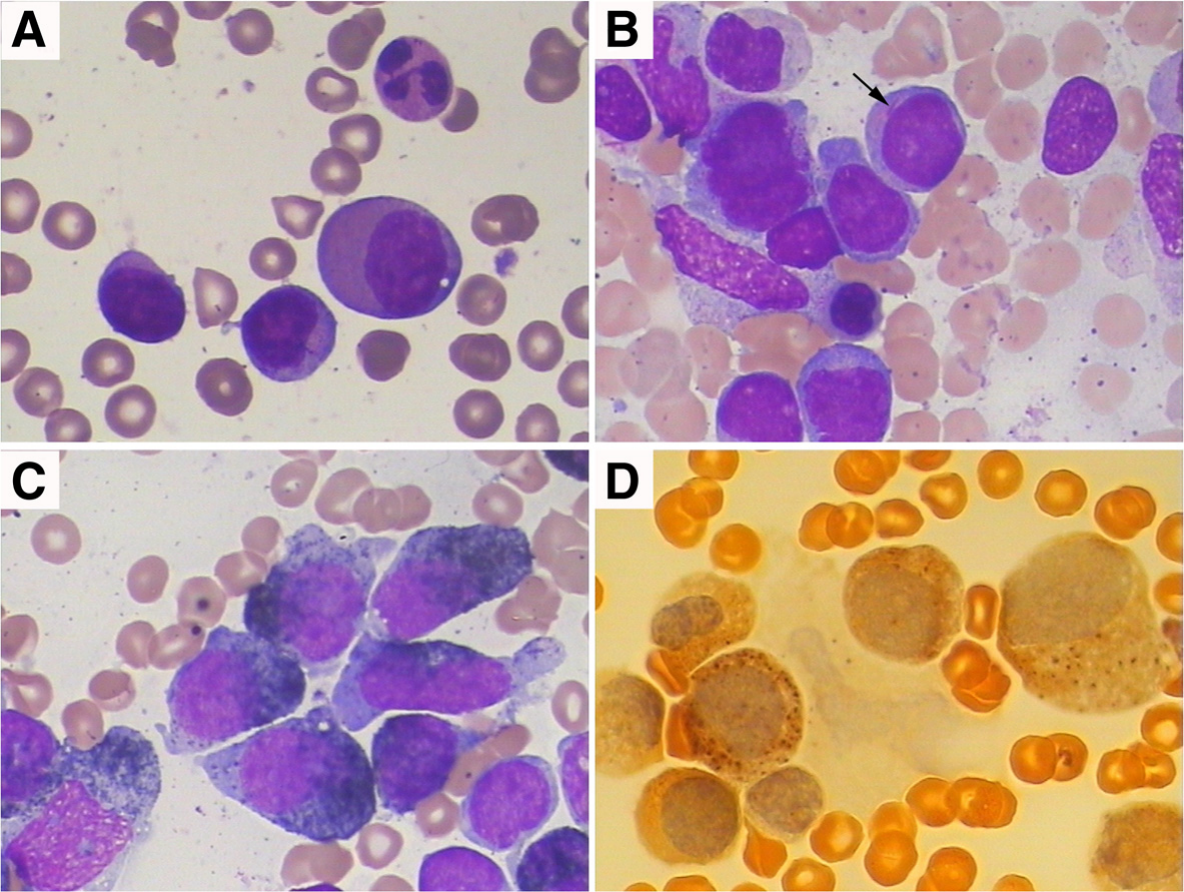
A smear preparation of a bone marrow aspirate in leukemic-phase in December, 2013. **a** The peripheral blood showed atypical blast cells with rich plasma, irregular nuclei and conspicuous nucleoli. The percentage of blast cells amounted to 41 % ( ×1,000, Wright–Giemsa stain). **b** Bone marrow was packed with pleomorphic large cells with abundant and basophilic cytoplasm containing some Auer bodies (×1,000, Wright–Giemsa stain). **c** These cells are positive for peroxidase staining (×1,000). **d** These cells are positive for non-specific esterase as monocytes (×1,000)

A 62-year-old Chinese man presented with gingival bleeding in August 2012. Physical examination revealed skin ecchymosis and enlarged lymph nodes in his cervical, axillary, and inguinal areas, the largest of which was 3 × 2 cm. Neither hepatosplenomegaly nor splenomegaly was noted. A complete blood count showed pancytopenia with a hemoglobin level of 11.1 g/dL, white blood cell (WBC) count of 2.92 × 10^9^/L (47.9 % neutrophils, 1.7 % monocytes, and 47.2 % lymphocytes), and platelet count of 21 × 10^9^/L. A bone marrow (BM) aspirate was hypercellular with 75.5 % myeloid cells, 19.5 % erythroid cells, and 4.5 % lymphocytes. Abnormal hematopoiesis was present in the granulocyte, erythroid, and megakaryocytic series to different extents, with 3 % blasts (Fig. 1a). However, flow cytometry (FCM) immunophenotyping of the BM showed no obvious abnormalities. A BM cytogenetic study revealed a 46, XY karyotype. Fluorescence in situ hybridization of the BM showed no myelodysplastic syndrome-associated karyotype [i.e., del (5q), del (20q), or +8 or del (7q)]. Total-body FDG-PET imaging revealed systemic lymphadenopathy (maximum standardized uptake value of 6.4). A right cervical lymph node biopsy showed disrupted node structure with effacement by diffuse distribution of pleomorphic neoplastic cells, most of which were large, and round to oval in shape. Some neoplastic giant cells were observed. The cytoplasm was usually abundant, and the nuclei were generally large and round with a prominent nucleolus. The chromatin was hyperchromatic, coarse, and granular. Mitotic figures were easily observed. Remaining lymphoid follicles were found. IHC phenotypes showed the following results: CD68++, MAC387++, lysozyme+, vimentin+, CK–, CD4–, CD5–, CD10–, CD15–, CD21–, CD23–, CD35–, CD30–, CD38–, CD138–, CD163–, CD56–, ALK–, CD79a–, CD1a–, TDT–, myeloperoxidase–, LCA–, CD20–, CD3–, HMB45–, S-100–, and Ki67 25 % (Fig. 2). The patient was therefore diagnosed with HS. He received systemic chemotherapy (CHOP regimen), consisting of vindesine, cyclophosphamide, and epirubicin on day 1 and prednisone on days 1–5. After 2 courses of chemotherapy, the enlarged cervical lymph nodes appeared to shrink and his peripheral blood parameters improved. After 4 courses, FDG-PET reexamination showed partial remission; BM showed disappearance of abnormal hematopoiesis, although 2 % blasts remained (Fig. 1b). As the patient refused to undergo stem cell transplantation, he underwent 4 additional courses of chemotherapy. His lymphadenopathy and pancytopenia recurred in March 2013, just a month after his last chemotherapy dose. The patient refused to undergo another lymph node biopsy. He was diagnosed with recurrent HS and treated successively with ICE, GDP, and Dexa-BEAM chemotherapy regimens, during which his peripheral WBC and blasts in BM gradually increased, although his lymph nodes shrunk. He was hospitalized again with body aches in December 2013. A complete blood count showed leukocytosis (WBC count: 50.72 × 10^9^/L; hemoglobin: 9.9 g/d; platelets 49 × 10^9^/L; 41 % blasts), and BM showed hypercellularity with 50 % blasts, the cytoplasm of which contained many Auer bodies, whereas the erythroid and megakaryocytic series were suppressed. These blast cells were positive for peroxidase and non-specific esterase (Fig. 3); periodic acid-Schiff staining showed fine grains. FCM analysis showed abnormal cells positive for CD117, CD13, CD33, HLA-DR, CD34, CD11c, CD38, and myeloperoxidase, but negative for CD68. Based on the morphologic and FCM findings, the patient was diagnosed with AMoL-M5, and treated with low-dose cytarabine, aclarubicin, and granulocyte colony-stimulating factor (CAG) regimen combined with decitabine. He died of severe pneumonia and hepatic failure in May 2014.

## Discussion

HS is an extremely rare malignant neoplasm. Tumors can occur over a wide range of ages (median age: 46 years), and is more common in males [17]. Clinical presentation can vary greatly, from isolated lymph node involvement to rapidly progressive disseminated extranodal disease. The gastrointestinal tract, spleen, soft tissue, and skin are the most common extranodal sites involved, although other locations have been infrequently reported [17]. Some patients also show systemic symptoms such as fever, fatigue, night sweats, and weight loss. Cytopenias are reported in about 30 % of patients with HS [18]. Generally, most patients with disseminated disease die within 2 years, whereas those with localized disease may benefit from surgery or chemotherapy (with or without radiotherapy), and thus have a relatively indolent clinical course [19].

The WHO classification strictly specifies the origin of the HS neoplastic cells to exclude diseases originating from cells other than histiocytes/macrophages, such as Langerhans cell tumor, follicular dendritic cell sarcoma, non-Hodgkin lymphoma, malignant melanoma, and monocytic leukemia. These neoplasms are mainly differentiated by IHC phenotypes, as doing so through morphology alone is difficult. Among them, HS and monocytic leukemia have some similarities in their IHC phenotypes, as both can express one or more monocytic neoplasm antigens, such as CD68, CD163 and lysozyme. However, AMoL, but not HS, commonly also expresses myeloid markers, such as myeloperoxidase, CD13, CD33, CD117. Myeloid marker expression is therefore the key to differentiating these two neoplasms.

Although HS is a rare disease, many reports in recent years have described HS arising subsequent to or concurrent with other neoplasms [3–13], most of which are lymphocytic leukemia/lymphoma. As HS coexists so commonly with other neoplasms, many hypotheses have been proposed regarding the mechanism of HS development. The first form is genuine HS, which is the malignant transformation of tissue histiocytes and apparently includes most cases of isolated HS. The second form is transdifferentiated HS, which results from transdifferentiation from other lineage neoplasms. This mechanism can be deduced from studies that showed the coexistence of HS with follicular lymphoma [3], B-cell acute lymphoblastic leukemia [8, 9, 11], and chronic lymphocytic leukemia [12], in which the HS was shown to share molecular or cytogenetic features with associated neoplasms. The third form is HS arising from pluripotential germ cells, which has been proposed because HS can occur in patients with mediastinal germ cell tumors [14, 15]. Only one case of HS to date was reportedly associated with monocytic leukemia. Mori et al. [13] described a 70-year-old man with peri-vertebral tumors and peripheral monocytosis of >1.0 × 109/L. Pathologic findings from a needle-biopsied tumor specimen supported the diagnosis of HS. However, the BM aspirate showed that both HS and CMML cells carried karyotypic abnormalities involving chromosome 8, indicating the existence of a common genetic background for both of them; this study proposed a new form of HS, termed transformation from monocytic neoplasm. Doll et al. [20] also reported a case of chronic myelomonocytic leukemia terminating as malignant histiocytosis, but IHC testing was not sufficient to prove the diagnosis of HS.

To our knowledge, AMoL has not been among the tumors reportedly associated with HS. In the present case, the initial lymph node biopsy showed typical morphological and immunophenotypic features of HS. Neoplastic histiocytes were positive for more than one histiocytic marker, including CD68, MAC387, and lysozyme. The patient’s specimens were typically devoid of Langerhans cells, follicular dendritic cells, specific B- and T-cell markers, and myeloid lineage markers, thus indicating a definitive diagnosis of HS. Lymphadenopathy and pancytopenia reoccurred 7 months later, and then leukocytes increased gradually. The BM showed the blasts had typical Auer bodies, positive for peroxidase and non-specific esterase. FCM analysis of BM also showed these cells to be positive for myeloid markers, thus establishing diagnosis of AMoL. Although no biopsy data showed whether the recurred lymphadenopathy was caused by HS relapse or to AMoL infiltration, the diagnosis of two neoplasms for the patient was clear from the typical manifestation of the primitive lymph node pathologic findings and the subsequent morphology, IHC phenotypes and FCM findings from the BM sample.

Monocyte/macrophage series derived from hematopoietic stem cells, which produce promonocytes that mature in the BM to monocytes, briefly circulate in the blood, and then enter tissues as macrophages to complete the maturation process [21]. If a patient suffers two homogenous neoplasms, HS and AMoL in a short period of time,we suspect that a promonocyte or stem cell, the upstream of both histiocytes and monocytes,might have malignantly transformed. If so, the mechanism is different from HS transformed from monocytic neoplasm proposed by Mori et al. [13], because HS occurred not after, but before, or at least simultaneously with the AMoL.

Unfortunately, we had not acquired cytological and molecular data from the lymph node and BM to prove the association of the two neoplasms directly. Moreover, CD68, one of the common monocyte/macrocyte antigens, was incongruous in the primitive lymph node IHC phenotypes and the FCM of leukemic-phase BM. The discrepancy might indicate these two neoplasms unrelated, but could also be due to a subtle distinction between HS and AMoL in CD68 expression (HS: ~100 %; AMoL: relatively lower) because of different differentiation stages [18]. We regret not being able to perform a bone marrow biopsy in the leukemic phase, as expression of histiocyte/macrophage markers, such as Mac 387 and lysozyme, would have shed some light here. Nevertheless, the potential association of both neoplasms in the present case couldn’t be eliminated.

The rarity of HS makes it difficult to establish standard treatment. Surgical resection with or without radiation therapy is the main treatment for localized disease and CHOP is often used as first-line chemotherapy for HS patients with advanced disease [19]. However, HS patients often relapse after CHOP and may require additional therapies, such as second-line chemotherapy and autologous hematopoietic stem-cell transplantation [22–24]. Additionally, thalidomide [23, 24], alemtuzumab (an anti-CD52 monoclonal antibody) [25] and the other novel targeted therapies including imatinib, sorrafenib and bevacizaumab [26] had also been reported to be therapeutic; however, the prognosis is generally poor. The patient with a combination of HS and CMML [13], and the present case, both had transient remission after chemotherapy. Development of an effective new therapy will be challenging.

## Conclusions

To our knowledge, this is the first case of HS combined with AMoL. The coexistence of these two neoplasms can be proved by the typical biopsy findings of lymph node and the myeloid markers of BM. The patient had a transient response to the chemotherapy and a poor prognosis. Whether these two homogenous neoplasms were inherently related is unclear. If so, we suspect this combination might be caused by malignant transformation of a promonocyte or stem cell, upstream of the histiocyte and monocyte.

## Consent

Written informed consent was obtained from the immediate family members of the patient for publication of this Case Report and any accompanying images. A copy of the written consent is available for review by the Editor-in-Chief of this journal.

## Abbreviations

AMoL: Acute monocytic leukemia
HS: Histiocytic sarcoma
BM: Bone marrow
FCM: Flow cytometry
CD: Cluster of differentiation
FDG-PET: Fluorodeoxyglucose-positron emission tomography imaging
IHC: Immunohistochemical
WBC: White blood count
WHO: World Health Organization
CMML: Chronic myelomonocytic leukemia

## References

[1] Grogan TM, Pileri SA, Chan JKC, Weiss LM, Fletcher CDM, Swerdlow SH CE, Harris NL (2008). Histiocytic sarcoma. WHO Classification of Tumours of Haematopoietic and Lymphoid Tissues.

[2] Weiss LM, Grogan TM, Mueller-Hermelink H-K, Jeff ES, Harris NL, Stein H, Vardiman JW (2001). Histiocytic sarcoma. World Health Organization classification of tumors: pathology and genetics of tumors of haematopoietic and lymphoid tissues.

[3] Feldman AL, Arber DA, Pittaluga S, Martinez A, Burke JS, Raffeld M (2008). Clonally related follicular lymphomas and histiocytic/dendritic cell sarcomas: evidence for transdifferentiation of the follicular lymphoma clone. Blood.

[4] Zhang D, McGuirk J, Ganguly S, Persons DL (2009). Histiocytic/dendritic cell sarcoma arising from follicular lymphoma involving the bone: a case report and review of literature. Int J Hematol.

[5] Hure MC, Elco CP, Ward D, Hutchinson L, Meng X, Dorfman DM, Yu H (2012). Histiocytic sarcoma arising from clonally related mantle cell lymphoma. J Clin Oncol.

[6] Alvaro T, Bosch R, Salvado MT, Piris MA (1996). True histiocytic lymphoma of the stomach associated with low-grade B-cell mucosa-associated lymphoid tissue (MALT)-type lymphoma. Am J Surg Pathol.

[7] Wang E, Papalas J, Hutchinson CB, Kulbacki E, Huang Q, Sebastian S (2011). Sequential development of histiocytic sarcoma and diffuse large b-cell lymphoma in a patient with a remote history of follicular lymphoma with genotypic evidence of a clonal relationship: a divergent (bilineal) neoplastic transformation of an indolent B-cell lymphoma in a single individual. Am J Surg Pathol.

[8] McClure R, Khoury J, Feldman A, Ketterling R (2010). Clonal relationship between precursor B-cell acute lymphoblastic leukemia and histiocytic sarcoma: a case report and discussion in the context of similar cases. Leuk Res.

[9] Bouabdallah R, Abena P, Chetaille B, Aurran-Schleinitz T, Sainty D, Dubus P (2001). True histiocytic lymphoma following B-acute lymphoblastic leukaemia: case report with evidence for a common clonal origin in both neoplasms. Br J Haematol.

[10] Castro EC, Blazquez C, Boyd J, Correa H, de Chadarevian JP, Felgar RE (2010). Clinicopathologic features of histiocytic lesions following ALL, with a review of the literature. Pediatr Dev Pathol.

[11] Kumar R, Khan SP, Joshi DD, Shaw GR, Ketterling RP, Feldman AL (2011). Pediatric histiocytic sarcoma clonally related to precursor B-cell acute lymphoblastic leukemia with homozygous deletion of CDKN2A encoding p16INK4A. Pediatr Blood Cancer.

[12] Shao H, Xi L, Raffeld M, Feldman AL, Ketterling RP, Knudson R (2011). Clonally related histiocytic/dendritic cell sarcoma and chronic lymphocytic leukemia/small lymphocytic lymphoma: a study of seven cases. Mod Pathol.

[13] Mori M, Matsushita A, Takiuchi Y, Arima H, Nagano S, Shimoji S (2010). Histiocytic sarcoma and underlying chronic myelomonocytic leukemia: a proposal for the developmental classification of histiocytic sarcoma. Int J Hematol.

[14] Song SY, Ko YH, Ahn G (2005). Mediastinal germ cell tumor associated with histiocytic sarcoma of spleen: case report of an unusual association. Int J Surg Pathol.

[15] Ladanyi M, Roy I (1988). Mediastinal germ cell tumors and histiocytosis. Hum Pathol.

[16] Fukunaga M, Kato H (2004). Histiocytic sarcoma associated with idiopathic myelofibrosis. Arch Pathol Lab Med.

[17] Takahashi E, Nakamura S (2013). Histiocytic sarcoma : an updated literature review based on the 2008 WHO classification. J Clin Exp Hematop.

[18] Pileri SA, Grogan TM, Harris NL, Banks P, Campo E, Chan JK (2002). Tumours of histiocytes and accessory dendritic cells: an immunohistochemical approach to classification from the International Lymphoma Study Group based on 61 cases. Histopathology.

[19] Hornick JL, Jaffe ES, Fletcher CD (2004). Extranodal histiocytic sarcoma: clinicopathologic analysis of 14 cases of a rare epithelioid malignancy. Am J Surg Pathol.

[20] Doll DC, Grogan TM, Greenberg BR (1987). Chronic myelomonocytic leukemia terminating as malignant histiocytosis. Hematol Pathol.

[21] Cline MJ (1975). The White Cell.

[22] Abu-Sanad A, Warsi A, Michel RP, Nahal A, Popradi G, Storring JM (2012). Long-term remission after autologous stem-cell transplantation for relapsed histiocytic sarcoma. Curr Oncol.

[23] Gergis U, Dax H, Ritchie E, Marcus R, Wissa U, Orazi A (2011). Autologous hematopoietic stem-cell transplantation in combination with thalidomide as treatment forhistiocytic sarcoma: a case report and review of the literature. J Clin Oncol.

[24] Abidi MH, Tove I, Ibrahim RB, Maria D, Peres E (2007). Thalidomide for the treatment of histiocytic sarcoma after hematopoietic stem cell transplant. Am J Hematol.

[25] Shukla N, Kobos R, Renaud T, Teruya-Feldstein J, Price A, McAllister-Lucas L (2012). Successful Treatment of Refractory Metastatic Histiocytic Sarcoma With Alemtuzumab. Cancer.

[26] Schlick K, Aigelsreiter A, Pichler M, Reitter S, Neumeister P, Hoefler G (2012). Histiocytic sarcoma - targeted therapy: novel therapeutic options? A series of 4 cases. Onkologie.

